# Alcohol and the Risk of Injury

**DOI:** 10.3390/nu13082777

**Published:** 2021-08-13

**Authors:** Tanya Chikritzhs, Michael Livingston

**Affiliations:** 1 National Drug Research Institute, Faculty of Health Sciences, Curtin University, Perth, WA 6008, Australia; michael.livingston@curtin.edu.au; 2 Centre for Alcohol Policy Research, La Trobe University, Bundoora, Melbourne, VIC 3086, Australia

**Keywords:** alcohol, injury, review, risk, mortality, morbidity, policy, intervention

## Abstract

Globally, almost four and a half million people died from injury in 2019. Alcohol’s contribution to injury-related premature loss of life, disability and ill-health is pervasive, touching individuals, families and societies throughout the world. We conducted a review of research evidence for alcohol’s causal role in injury by focusing on previously published systematic reviews, meta-analyses and where indicated, key studies. The review summarises evidence for pharmacological and physiological effects that support postulated causal pathways, highlights findings and knowledge gaps relevant to specific forms of injury (i.e., violence, suicide and self-harm, road injury, falls, burns, workplace injuries) and lays out options for evidence-based prevention.

## 1. Introduction

Globally, almost four and a half million people died from injury in 2019 [1], with 7% of these deaths directly attributable to alcohol. Alcohol’s role in injury-related premature loss of life, disability and ill-health is pervasive, touching individuals, families and societies the world over. Alcohol use, particularly intoxication, plays a major role in a wide range of injuries, some of which are readily recognisable as alcohol-related (e.g., road injuries, violent assault) and others which are less so (e.g., falls, drownings, injuries in the workplace).

Alcohol-attributable injury accounts for around one-tenth of the total impact of alcohol on health (9.9% and 12.6% in low- and high-income countries, respectively) [1]. Males (90%) and young people aged 15–39 years (40%) dominate alcohol-attributable injury deaths [1]. Impacts on health systems are considerable, with alcohol contributing to between 5% and 40% of all emergency department (ED) injury presentations across 27 countries [2]. This comes with significant costs. In 2014, injuries caused by alcohol in the USA were an estimated 8% of all injury-related ED presentations at a cost of nearly USD 9 billion, and when in-patient admissions were added, costs almost tripled (USD 26 billion) [3]. Canadian estimates of hospitalisation and day surgery costs for alcohol-attributable injuries in 2017 were just under CAD 1 billion [4]. Large economic impacts are not limited to high-income countries. In Sri Lanka, alcohol-attributable injury costs exceeded an estimated USD 380 million, nearly half of the total costs of alcohol in that country in 2015 [5]. In Latin America, where around 30% of road fatalities are attributable to alcohol [6], the burden of road crashes overall was between 1.5% and 3.9% of gross domestic product in 2013 compared to about 2% in the USA [7].

Alcohol-related injuries thus represent a significant economic burden in many societies globally and require substantial resources from overstretched health systems to manage. Importantly though, alcohol-related injuries are preventable and there are clear examples of effective interventions to reduce them. For instance, deaths due to drink-driving declined rapidly in many high-income countries during the 1980s [8,9], driven by tougher drink-driving laws and enforcement alongside broader alcohol policy shifts such as increases in the legal minimum drinking age. However, from a global perspective, rates of overall alcohol-related injury have remained largely stable over time [1] and are likely to increase in coming years as alcohol consumption in lower- and middle-income countries increases [10].

This review will summarise research evidence linking alcohol to physical injuries, including potential causal mechanisms involved. The review also highlights key research findings specific to various forms of injury and lays out options available for prevention. Given the breadth of information we aim to cover here, we take a narrative review approach, relying largely on previously published systematic reviews, meta-analyses and focusing on key studies where appropriate.

## 2. Alcohol as a Cause of Physical Injury

Research evidence for alcohol as a cause of injury has clearly emerged for many types of injury, across multiple settings, and using a wide range of study designs. Key forms of research evidence include: laboratory experiments conducted under controlled conditions [11], real-world emergency department studies [12], driving simulation studies [13], studies linking population level drinking and injury rates [14] and retrospective time-series studies showing that alcohol policy changes and interventions can influence population rates of injury [15].

In one of the most comprehensive reviews of individual-level data, Taylor et al. [16] demonstrated strong dose–response relationships between amount of alcohol consumed in the past 3 h and odds of both motor vehicle and non-motor vehicle injury. Their meta-analysis estimated that even relatively moderate consumption levels (24 g of pure alcohol) roughly doubled the odds of injury, but that risks increased sharply at higher levels of consumption, such that someone who had consumed 120 g of alcohol had a more than 50 times higher risk of a motor vehicle injury than a non-drinker. Other reviews have shown that these effects are broadly consistent across different study designs and alcohol recall periods, suggesting robust relationships [17,18].

At the population level, time-series analyses have shown that changes in per-capita alcohol consumption are associated with changes in mortality rates related to road injuries [19], suicide [20] and homicide [21,22]. These studies (see [14]) clearly showed that the amount of alcohol consumed in a given society is a key driver of injury rates, although there is significant variation cross-nationally, reflecting variation in drinking patterns and prevention policies at the country level. These established relationships between alcohol use and physical injury have underpinned regulation and public policy in many countries. Some applications, such as legal blood alcohol limits for driving [23], reach back many decades, while others, such as integration into national drinking guidelines [24], are relatively recent.

### Plausible Causal Pathways: Pharmacological and Physiological Actions of Alcohol on the Human Brain and Central Nervous System

Alcohol is a known neurotoxin and central nervous system depressant. Even at low to moderate levels, alcohol has been observed to impair balance, visual focus, reaction time, judgment and to change behaviour (e.g., [25]). At high enough doses, intoxication can result in loss of consciousness, coma, respiratory failure (i.e., due to airway obstruction), aspiration pneumonia and ultimately, death [26].

Regarding plausible causal pathways that explain links between alcohol and injury, experimental studies offer the firmest evidence by virtue of their ability to randomly assign participants to placebo and exposure groups, subjectively measure functional biomarkers and control alcohol dosage. Though not immune, experimental studies are also best equipped to separate out pharmacological/physiological effects from ‘expectancy’ effects, i.e., personal beliefs about how alcohol affects behaviour, such as physical aggression, which can vary widely among individuals and cultures [27,28].

Laboratory studies which test human performance on various tasks designed to detect alcohol effects on specific brain systems have identified substantial impairments across multiple measures of cognitive (e.g., information processing) and psychomotor functions (e.g., eye-brain-hand-foot coordination) that directly bear on all forms of injury risk. An extensive review of more than 200 controlled experimental studies on alcohol’s acute effects on the brain and central nervous system found impairments for visuo-motor control, divided attention, focused attention, reaction time, response inhibition and working memory. Effects were highly consistent at blood/breath alcohol concentrations (BACs) of 0.05% and higher, and some effects were found even at lower levels [29].

Extending what has been learnt from standard laboratory experiments, in vivo neuroimaging studies can detect alcohol’s pharmacological effect on the human brain and bring potential mechanisms for alcohol-caused injury into clearer focus. Reviews of neuroimaging studies consistently support findings of diminished cognitive and psychomotor functions identified by laboratory experiments [28]. Several reviews, incorporating studies with a wide range of designs, show that beginning at low levels, acute alcohol intake reduces overall brain glucose metabolism (a proxy for neuronal activity) and increases metabolism of acetate (a product of acetaldehyde oxidation) in a dose–response manner. Reduced glucose metabolism is most concentrated in the cerebellum (implicated in motor impairment), while limbic regions (implicated in reward-seeking behaviour and addiction) show increased metabolism [28,30,31]. These studies also show that brain centres most affected by alcohol (i.e., cerebellum, hippocampus, occipital cortex, striatum, amygdala) are regions where balance, movement coordination, attention focus, self-control, processing of emotional stimuli (e.g., threat detection), motivation and reward-seeking, spatial learning and memory are believed to occur.

There is strong concurrence, therefore, between reviews of experimental laboratory studies demonstrating cognitive and performance deficits and neuroimaging studies demonstrating pharmacological and physiological actions of alcohol on the brain that strongly implicate causal pathways to injury risk. It is crucial, however, to bear in mind that the relationship between alcohol and injury is by no means inexorable. Outside of the laboratory, observational studies confirm every-day experience that not all alcohol use, or even intoxication, necessarily results in injury. Risk of injury from alcohol can be influenced by individual differences and expectancies about appropriate or permissible behaviours [27,32,33] as can social and cultural norms (e.g., community acceptance or rejection of drinking and driving). External factors such as setting (e.g., home, pub, park), price and physical availability of alcohol also have major impacts on alcohol-caused injuries at a population level [34,35].

## 3. Specific Injury Types

The injury literature often distinguishes between injuries arising from intentional behaviours and those that are most often unintentional or accidental. Interpersonal violence, self-harm and suicide are all considered intentional injuries as they arise from purposeful actions directed towards oneself or others. Unintentional injuries on the other hand include road injuries, other transport injuries, falls, drownings, burns, poisonings, workplace injuries and other ‘accidents’ (e.g., freezing), and are often further categorised into transport and non-transport. Categorizing injuries according to intention is commonplace in the literature and assumed to have utility for injury management and prevention. For instance, prevention approaches to intentional injuries often focus on characteristics of individuals and their behaviours, while unintentional prevention initiatives are more often concerned with how people, objects and environments interact [36]. It is nevertheless worth noting that some have questioned whether categorisation limits collaboration and advancement of prevention efforts, as the underlying motivations of individuals are not always clear-cut (e.g., some burns are intentional, as are some road injuries), and groups share many similar characteristics, including effective prevention approaches (see Section 4 on policy and interventions below) [37].

**Figure 1:**
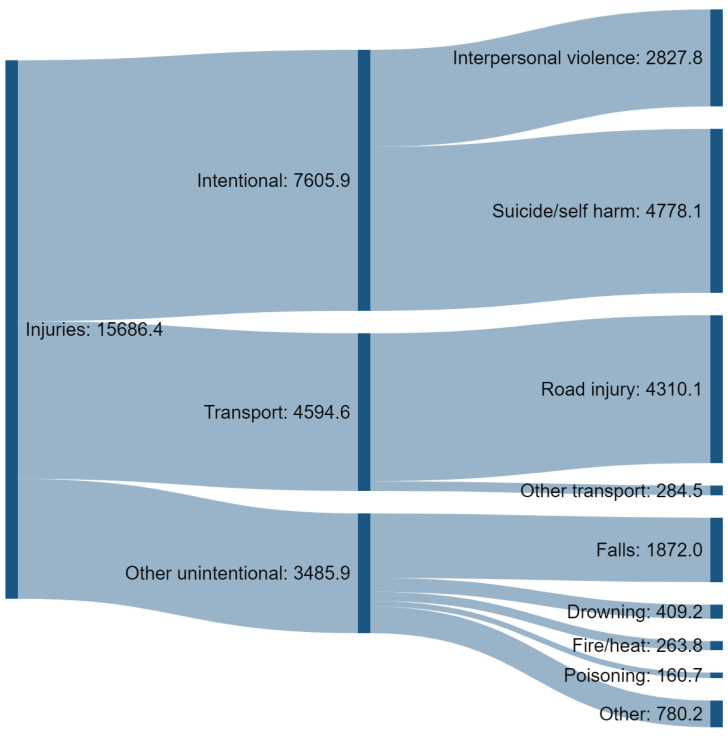
Global Disability Adjusted Life Years (DALYs ’000) lost due to alcohol-attributable injuries, 2019. Data source: Institute for Health Metrics and Evaluation [1].

In terms of morbidity and mortality, alcohol-attributable intentional and unintentional injuries each account for roughly 50% of total numbers of Disability Adjusted Life Years (DALYs), Deaths and Years of Life Lost (YLL), with transport (i.e., mostly road injury) making up the majority of unintentional injuries [1]. Figure 1 presents the estimated global impact of alcohol-related injury in terms of Disability-Adjusted Life Years (DALYs), breaking down the impact into the broad categories of injury.

### 3.1. Interpersonal Violence

Interpersonal violence (IV) refers to intentional use of physical force (sexual or non-sexual) by an individual or small group of individuals against another individual or small group, and excludes larger scale conflict-related violence (e.g., warfare, rioting) [38]. In addition to sexual and/or non-sexual physical aspects, IV may also involve deprivation, neglect and psychological aspects. In the past decade or so, there has been increased focus on IV as a global problem posing major challenges to sustainable development goals including poverty, health and wellbeing, human rights and gender equality, particularly for women and girls (e.g., EU and UN Spotlight Initiative [39]).

Findings from many meta-analyses, some of which focused on laboratory studies (e.g., [40]), others on community-based studies (e.g., [41]), and even a recent meta-meta-analysis of 18 reviews covering multiple designs, settings and definitions of violence [42], strongly support alcohol use, especially by males, as a causal factor in IV. Although at times controversial, there is also robust evidence supporting the conclusion that alcohol use by victims at the time of the offence increases the risk of IV [42,43]. The role of alcohol use by females and IV has been less well-studied than for males, however, female alcohol use has also been identified as a risk factor for both perpetration and victimisation. Moreover, alcohol use is more strongly linked to victimisation among women than victimisation among men in intimate partner violence (IPV) [44,45].

Alcohol use by parents and caregivers, particularly at harmful or hazardous levels, also increases the risk of child physical injury, such as burns, fractures and, occasionally, death, arising from maltreatment [46]. Heavy alcohol use by either victim or perpetrator is also a risk factor for the physical abuse of older people by offspring, partners or other relatives in a caregiver role, as well as professional caregivers to a lesser extent [47].

Global burden of disease estimates indicate that as a proportion of total alcohol-attributable injuries, IV accounts for about 16% of deaths and 18% of DALYs [1]. However, IV is highly prone to under-reporting in official statistics, often as a result of victims avoiding authorities or official agencies for fear of further victimisation. In addition, effects of IV can extend well beyond immediate physical consequences that might compel a victim to seek treatment (e.g., emergency department attendance) or formal assistance (e.g., police, social services). Experience of trauma for instance, especially at a young age, increases risks of developing mental health problems, reproductive and sexual health problems, substance misuse, chronic illness (e.g., cardiovascular disease, diabetes, cancer) and of living in poverty later in life, very little of which is readily quantifiable [48].

### 3.2. Suicide and Self-Harm

Suicide and self-harm are second only to road injuries in terms of injury-related burden of disease contribution [49] and a major cause of death for young people [50]. The GBD estimates that around 15% of all suicide deaths are attributable to alcohol, meaning over 100,000 people die each year from alcohol-related self-harm. A series of systematic reviews have found strong and consistent evidence that alcohol and self-harm are strongly linked. This includes individual-level studies showing that people with alcohol use disorders (AUDs) are at increased risk of suicidal ideation, self-harm and completed suicide [51], studies showing that heavy drinking in-the-event increases suicide risk [52] and aggregate studies showing population-level links between alcohol consumption and suicide rates [20]. Evidence also points to violent methods of suicide, such as by firearm or hanging, involving heavier drinking in-the-event compared to poisoning (e.g., [53,54]).

Alcohol’s role in self-harm appears to be mediated through cultural factors—for example, there is some evidence that alcohol is more strongly associated with self-harm for men and for cultures where intoxication-oriented drinking is more common [20]. Further, while the evidence is reasonably clear that alcohol contributes to suicide and self-harm, there remains uncertainty about the magnitude of the causal relationship, with at least some potential for alcohol use disorder, intoxication and self-harm to have common underlying drivers [20].

### 3.3. Road Injuries

Road injuries typically include fatal and non-fatal injuries that occur on public roads as a result of accidents involving one or more motor vehicles (e.g., cars, motorcycles, trucks), pedestrians or cyclists. Road injuries are currently ranked 7th for their contribution to total global DALYs (2.9%) for all ages [55]. Beginning in about 1980, many high-income countries reported substantial reductions in road injury rates. Rapid declines in national road tolls continued for about 15 years, largely as a result of concerted prevention efforts including legislation and enforcement of maximum legal blood alcohol concentration levels for driving [9]. After this time, improvements in high-income countries slowed considerably (e.g., [56]), although downward trends continued on a global scale with age-standardised DALYs for road injury declining for all ages (31%), 10–24 year olds (33.6%) and 25–49 year olds (22.5%) [55] between 1990 and 2019. Even so, worldwide, road injury remains the leading cause of death and disability for 10–24 year olds (6.6%) and 25–49 year olds (5.1%) [55].

Global statistics obscure large differences in road injury rates across nations and regions, particularly between high-income and low to low/middle-income nations. In the African region for instance (where under-reporting is widespread) [57], road fatality rates lead the world, are at least double that for the European region [58] and have shown only marginal improvement over recent decades [57]. This suggests that a great deal of road injury prevention work remains to be carried out, particularly in Africa and South-East Asia where road safety laws, including for drink-driving, rarely meet best-practice standards [58].

The causal, dose-dependent role that alcohol plays in fatal and non-fatal road crashes has been well-established over decades of extensive observational, laboratory and driving simulation research [13,16,59]. Alcohol has been shown to impair driving performance at blood alcohol concentrations as low as 0.02% [60,61] and well before the driver or observers are able to detect signs of intoxication [62]. With a few notable exceptions (e.g., Sweden [63]), most countries maintain maximum legal blood alcohol concentration levels for non-probationary drivers (e.g., 0.05%, 0.08% [58]) that are higher than levels now known to significantly increase crash risk.

Alcohol-impaired drivers increase road injury risk to themselves and others, including passengers, pedestrians and other drivers [58,64]. Besides motor vehicle operators, alcohol-positive pedestrians [65] and cyclists [66,67] are also at increased risk of road injury. Drink-for-drink, young and inexperienced drivers are at much greater risk of serious road injury than their more experienced counterparts [56,61]. Among drivers with alcohol use disorder, road crash risk is at least twice that for non-dependent drivers (e.g., [68]).

Estimates of country-specific alcohol-attributable fractions for road fatalities vary considerably, however, averages for broad regions range from about 2% in the Eastern Mediterranean where alcohol consumption is largely prohibited, to almost 38% in Europe where alcohol is widely available [6]. Among all alcohol-attributable injuries, road injuries account for over one quarter (27.5%) of total DALYs and they also account for more than half of all unintentional injuries (see Figure 1).

### 3.4. Falls

Falls represent a major contributor to morbidity and mortality, ranking 21st across all ages (second only to road injuries in terms of injury) and 8th for people aged 75 years and older as a cause of age-standardised DALYs [55]. Not surprisingly, for those aged over 70, falls are the most common cause of an injury-related death [49].

Systematic reviews of studies that examine usual drinking practices and fall risk generally produce mixed results [69,70], however the majority fail to account for patterns of drinking. In a pooled analysis of case-control studies from emergency departments in 28 countries, Cherpitel et al., showed that both frequent and episodic heavy drinking were strong predictors of alcohol-involved falls [71]. Studies that have examined drinking in-the-event are even more compelling. In a meta-analysis of five studies that used acute measures of drinking, Taylor et al. [16] found a clear dose–response relationship, with odds of a fall-related injury increasing by 1.15 for each 10 g of alcohol consumed. This is supported by other reviews [70] and pooled analyses of ED studies [12,72].

Recent work has identified concerns about interactions between alcohol consumption and use of medication among older populations, where falls represent a disproportionately large cause of morbidity [73]. This points towards one possible specific intervention to reduce the burden of falls—better assessment and management of alcohol consumption risks by primary healthcare workers when administering/prescribing medications, especially those related to the central nervous system [73].

Studies have repeatedly shown that accidental fall injuries result in substantial costs [74]—for example, a US study estimated the annual cost of fall injuries at more than USD 80 billion [75]. National populations throughout the world are ageing [76] and, although trends vary across countries, some studies have reported increasing levels of risky drinking among older age groups [77,78,79,80]. Alcohol-related falls are therefore likely to present an increasing social burden for many countries in coming decades.

### 3.5. Drowning

A recent systematic review [81] found that around half of all drowning deaths and more than one-third of all drowning-related injuries involved alcohol, but that the prevalence of alcohol involvement varied markedly between studies. The literature relies heavily on post-mortem assessments of BAC or relatively crude cross-sectional surveys, meaning the strength of the causal evidence is relatively low, but the best estimates for proportion of all drownings causally attributable to alcohol range from 10% to 30% [82], with people who have blood alcohol concentrations of 0.10% or higher increasing their risk of drowning ten-fold [82].

### 3.6. Injuries from Excessive Heat and Cold

Alcohol intoxication raises the risk of sustaining serious injuries from excessive heat, such as burns from household fires, and from excessive cold, such as hypothermia or death from freezing when drinking outdoors in cold weather. Systematic reviews consistently identify alcohol intoxication as a key risk factor for residential fire mortality [83,84], with around half of all house fire fatalities tested returning positive BACs [85]. In a robust US case-control study, presence of an intoxicated person in the household was the single strongest predictor of fire leading to fatality [86]. Alcohol intoxication delays escape and increases risk of fire ignition, particularly in conjunction with smoking (e.g., falling asleep while drinking and smoking) [87]. Acute and chronic heavy alcohol use, particularly among older age groups, are also major risk factors for serious hypothermia and death by freezing, although increased risk of hypothermia can also occur among the young (e.g., [88]). Likely under-reported at a global level, alcohol’s role in injuries arising from excessive cold nonetheless present ongoing challenges for cold climate countries during winter months, with many reporting alcohol’s involvement in more than 40% of fatal cases (e.g., [89,90,91]).

### 3.7. Workplace Injuries

Despite growing use of alcohol and drug testing in the workplace, international research evidence for alcohol as a major contributor to workplace accidents and injuries (except impaired driving) is surprisingly under-developed. Although single studies have continued to support a causal relationship [92,93,94], almost three decades have passed since Stallones and Kraus’ [95] review of epidemiological evidence regarding alcohol’s role in workplace injuries.

### 3.8. Alcohol Poisoning and Other Injuries from Heavy Intoxication

Other key forms of injury arising from heavy intoxication include aspiration (i.e., choking) [49] and alcohol poisoning [96]. These are especially common among marginalised populations in intoxication-oriented and spirits drinking cultures, with—for example—rates substantially higher in Eastern Europe than the rest of Europe [97]. Additional injury risks arise when informal (e.g., home-made) or illegally produced counterfeit or adulterated products are consumed. These products often contain unknown quantities of pure alcohol (i.e., ethanol) and other toxic substances (e.g., methanol, ethylene glycol) not intended for human consumption (e.g., ‘antifreeze’, perfume, methylated spirits) that can cause blindness, brain injury, coma and death when ingested [98,99]. People with alcohol use disorders, low incomes and tourists appear to be at particular risk of injury from illicit alcohol. Large poisoning outbreaks have been documented in many countries, with fatality rates as high as 30% in some places (e.g., Uganda, Tunisia, Turkey, Pakistan, Norway, Nicaragua, Libya, Kenya, Indonesia, India, Estonia, Ecuador, Czech Republic, Cambodia) [100,101].

## 4. Effective Interventions and Policies

Broadly speaking, research evidence for effective interventions and policies aimed at reducing alcohol-related injuries can be grouped into two camps: (i) alcohol consumption-centred approaches oriented towards reducing use at a whole-of-population level, that may also have specific or more substantive effects on sub-populations (e.g., young people, heavy drinkers), and (ii) injury-centred approaches targeted at reducing the risk of specific types of injury (e.g., falls, assaults) or injuries that occur in specific situations (e.g., while driving, in the workplace). Though not intended to be exhaustive, the following sections summarise current research evidence for a wide range of policy and intervention options available to decision makers concerned with reducing alcohol-related injury.

### 4.1. Alcohol Consumption-Centred Approaches

Decades of research evidence clearly support policy approaches that reduce population-level alcohol consumption as having a central role to play in the reduction of alcohol-related injury overall. Whole-of-population consumption-centred approaches are highly cost-effective at reducing harmful alcohol use in general, alongside restrictions on marketing and brief interventions [102]. Furthermore, although most evidence in support of consumption-centred approaches has been derived from high-income countries, they are also highly effective in middle- and low-income countries when implemented appropriately [103].

Among consumption-centred approaches, research evidence in support of effective reduction of population-level alcohol use is arguably the strongest and most consistent for price-based interventions that influence alcohol’s economic availability, i.e., retail price relative to disposable income [104,105,106,107]. A meta-analysis of 50 studies suggested that a doubling of alcohol taxes would reduce road injury deaths by 11%, violence by 2% and suicide by around 4% [108]. Price-based interventions have historically been delivered via governments raising alcohol taxes. However, there is growing evidence that raising the minimum price at which alcohol can be sold at retail (i.e., minimum unit pricing, MUP) is an effective strategy for reducing overall consumption, IV and drink-driving [109,110,111,112].

There is also very strong evidence that raising the legal minimum drinking age (e.g., from 18 to 21 years) leads to substantial reductions in road injuries among young people in the USA and elsewhere [113,114], and is most effective when supported by concerted enforcement efforts [115,116]. Benefits of higher legal minimum drinking age laws have also been shown to accrue to suicide [117], violence and morbidity from other accidental injuries [118] among young people.

Strategies which reduce alcohol’s physical availability can substantially reduce injury rates, including in low- and middle-income countries [119]. Research evidence is particularly robust for reductions in permitted hours of sale of alcohol late at night and reduced violence and road injuries [15,120]. Links between physical density of outlets (i.e., both on- and off-trade access) and injury outcomes (including violence, road injury and self-harm [121]) have been demonstrated by many studies. However, uncertainties remain about the overall robustness of this literature [122] and confirmation is needed from new studies that incorporate information on alcohol sales with appropriate methods for studying geospatial data.

Of recent interest are potential impacts from alcohol’s designation as an ‘essential’ product/service and liberalisation of off-trade alcohol sales by a large number of jurisdictions during the COVID-19 pandemic [123,124,125,126]. Expansion of off-trade sales often occurred simultaneously to the closure of workplaces, schools, childcare, leisure and physical activity centres [126]. Although evidence is still emerging, reports in the media and grey literature suggest increased drinking in the home in some countries [127,128,129] (largely off-setting reductions in on-premises drinking). These reports have appeared alongside several studies showing increased abusive head trauma among children [130] and family violence [131,132]. There is some concern that, pressured by commercial vested interests [133,134], governments will allow continuation of deregulatory changes originally intended as temporary, leading to increased risks of IV and trauma in the home [123,124,126].

Multi-component interventions that simultaneously implement a suite of strategies can markedly reduce injury, especially when supported by their target populations [135]. Although price increases and physical availability restrictions (e.g., reduced trading hours, limits on cheap high-risk beverage purchases) are considered central to the success of these programmes, they are often accompanied by supporting harm (e.g., mandatory server training, sobriety testing) and demand reduction strategies (e.g., advertising restrictions) [135,136]. Most recently, a series of price and availability restrictions in Lithuania reduced total mortality there by 3% [137], with the bulk of benefits arising from reductions in injury-related deaths [138]. Due to their relatively direct control of alcohol sales, advertising and promotion, jurisdictions with whole or partial alcohol monopolies (more common to Scandinavia and North America) are well-placed to implement multi-component interventions. All else being equal, alcohol monopolies have lower rates of alcohol-related injuries than those with free market systems [139]. Studies which have modelled potential impacts of disbanding retail monopolies in Sweden, for example, have estimated increases in alcohol-related injury deaths of between 18% and 28% annually [140].

### 4.2. Injury-Centred Approaches

From a global perspective, evidence-based strategies for reducing alcohol-related road injuries have undoubtedly received more government commitment to implementation than any other source of injury—and with striking results [58]. Of critical importance to minimizing the road toll in countries where alcohol is widely consumed are government laws prohibiting BACs exceeding 0.02% for probationary drivers and 0.05% for non-probationary drivers (WHO 2018). Current best-practice drink-driving laws should also be coupled with widely publicised, highly visible police enforcement and random breath testing [141]. At last count, 45 countries, covering less than a third of the world’s population, had enacted such laws, with just 2% from low-income countries [58]. There is great scope, therefore, for governments of countries at all income levels to substantially reduce premature death and disability caused by alcohol-impaired road users among their citizenry.

Motor vehicle drivers who repeatedly drink and drive are often targeted for further preventative measures. Commonly referred to as ignition- or alcohol-interlocks, devices that detect breath alcohol can be retrofitted to motor vehicles of drink-driving offenders. When positive breath alcohol is detected, alcohol-interlocks incapacitate a vehicle by blocking engine ignition and are highly effective at reducing repeat drink-driving offences [7,142].

Interventions aimed at reducing alcohol-related IV have largely been focused around drinking venues and night-time entertainment precincts. While broad physical availability restrictions (e.g., reduced trading hours for licensed venues) have the clearest evidence, improving server training [143], venue security, environmental design and management have been shown in some settings to reduce violence and aggression [144]. Research into interventions aimed at alcohol-related domestic or family violence specifically is relatively scant, with systematic reviews finding few robust evaluations of alcohol policy interventions [145]. Individual-level interventions with offenders have shown generally poor results in terms of reducing reoffending [146], suggesting that upstream policy interventions should be the focus of work to reduce alcohol-related violence.

Research on interventions to reduce alcohol-related drowning or burns is scarce. A study of minimum-legal drinking age laws in the USA found no impact on young-adult drowning rates [147], while programs aiming to reduce alcohol consumption while boating and fishing remain largely unevaluated [148]. Similarly, evaluation studies of workplace alcohol and drug testing are poor, with only one relatively high-quality study finding an effect of testing in the transport industry [149].

## 5. Conclusions

Invitations to consider alcohol’s role in the death, disability and distress that arises from human injury, and what actions could be taken to reduce the burden, can evoke a wide range of responses. To varying degrees, responses may be part of a broader agenda, compromised by politicisation, motivated by vested interests or simply reflect personal beliefs and experience. In contrast, scientific evidence in support of alcohol’s causal and central role in injury has strengthened over time and is strikingly robust. Given the broad range of scientific disciplines and research approaches which have contributed to the evidence base, the many decades over which that evidence has accumulated and the variety of forms that alcohol-related injury can take, the high level of overall consistency among findings is remarkable.

Estimates of the human and economic costs of alcohol-related injury leave no doubt that the global burden is very large. In truth, it is probably larger still, and it may be many more decades (if at all) before the full extent of short- and long-term consequences, including chronic disease, mental health problems and reduced wellbeing, are fully understood and quantified across all counties. At the very least, the burden borne by low- and middle-income countries is likely far higher than current statistics imply.

Upward global trends in per capita alcohol consumption during the past thirty years or so are predicted to continue, increasing by more than a litre per person by 2030 (i.e., from 6.5 L in 2017 to 7.6 L in 2030) [10]. Increasing consumption combined with ageing populations and more drinking in the home (facilitated by pandemic-related liberalisation of off-trade sales) can be expected to bring about changes in the distribution and magnitude of alcohol-attributable injury in the next several decades. These changes may well add to challenges faced by governments already struggling to manage over-loaded healthcare systems, policing and social services [150].

Nonetheless, there is reason to be optimistic. The evidence is clear: population-level alcohol consumption-centred policies that reduce alcohol’s economic and physical availability, especially when implemented in conjunction with each other, substantially reduce alcohol-related injury in its various forms. Strategies specifically targeted at reducing alcohol-impaired driving are also highly effective and indeed essential for addressing the world’s leading cause of death and disability among people in their most productive years. There are major human capital and economic windfalls awaiting governments that adopt nation-wide, best-practice alcohol interventions. Manifestly underutilised, independently and collectively, the full potential of these strategies has been scarcely realised, though they offer evidence-based solutions for high- and low-income countries alike [151].
